# A history of Plastic Surgery

**Published:** 2008-10

**Authors:** Ravin Thatte

**Affiliations:** 46, Shirish, 187, Veer Savarkar Marg, Mahim, Mumbai - 400 016, India. E-mail: mail@artmartindia.com

It was with considerable dread that I accepted an invitation to review this tome with its fine print and numerous, ancient illustrations. My feelings of inadequacy for this task arose mainly because of my temperament, which does not allow me to settle down to any kind of detail, or to delve in depth into any matter. However, as I undertook this rather unwelcome mission, the book took hold of me because it was not only a history of scientific endeavours, but also a chronicle of exceptional human intuitions as to how human nature unfolds as it struggles between dry scientific truth and personal vanity. The book cites innumerable instances of many talented, dedicated, and productive men with great ideas who could see that some of their methods were not succeeding but were loathe to admit it during their lifetime. Be that as it may, the majority of them were honourable scientists on whose shoulders we stand today. Moreover, the authors of this book have done a remarkable job of walking the tight rope of being neither too adulatory nor too critical, leaving the readers to judge for themselves. For those who are in the academic world or who are trying to fashion new techniques, this book carries a very important and also, subtle message.

Reading this book was also an eye opener for a person (like me) steeped in the British tradition of plastic surgery (in my formative years the Anglo was more prominent than American). It was for the first time that I was to become aware of the European contributions to plastic surgery, which obviously, are very formidable. This is not entirely my fault but it does have something to do with the political and colonial history of the world. This book, an anglo-European effort, will go a long way in restoring the balance, which to my mind, was lacking in the past for most nonEuropean plastic surgeons.

The authors state in their preface and I quote, “our specialty has become increasingly sophisticated in recent times….” Even at the cost of being branded pedantic, I quote the meaning of the word “sophisticated” as given in the Oxford dictionary, *i.e*., “educated, cultured, or refined, make (equipment or technique etc.) highly developed or complex, mislead (a person) by sophistry, deprive (a person or a thing) of its natural simplicity, make artificial by worldly experience, tamper with (a text etc.) for purpose of an argument, or adulterate (wine etc.)”. Which of these meanings the authors meant to use, is not known. The authors are charitable gentlemen, but obviously, are not enthusiastic about what has transpired in the recent or not too distant past, because it is in this latter (smaller) part of the book that it falls apart. The authors state that they use an indefinite article in the title of their book (“a”). But that is not justification enough to treat that part of the book cursorily. Some of the illustrations in this section make for horrible viewing as compared to the elegance and magnificence of the illustrations in a major portion of the book, and are like the Mona Lisa set next to a computer graphic. As to at least one meaning of the word “sophisticate”, what is one to say about our specialty (currently) which switches its loyalties in a matter of few years from a subperiosteal face lift (rejuvenation?!) by a bicoronal incision, to artificial threads under local anaesthesia and then claim that the threads are as effective if not better. This is commercial chicanery at its best (or worst) let loose on a population swimming merrily in sensorial anarchy, thanks to their disposable incomes. If another edition of this book was to come out, the authors should think about omitting this rather ugly and false tail from this otherwise graceful animal. True, our specialty has metaphorically shifted from an artistic studio to clever laboratories and repetitive technological workshops, but then that will require another book which will have to be written by someone who is more adept at looking at this mechanical world.

Having said that, this book is a treasure of a huge number of references, both published and held privately, and its arrangement is tantalizingly attractive. The text reads like fiction but is entirely historical, and is well supported by documented proof. The efforts taken by the authors must have been huge.

After reading the book meticulously, I found some grammatical errors in the text as well as some erroneous conclusions such as, “a truly successful vaginoplasty had to wait till a gracillis muscle flap was developed”. But while the former was expected because of the difficulty of the transition from one author and language to another, the latter is a symptom of the fact that the authors did not pour their hearts into this part of the book. Judging by their preface, I am sure they are conscious of this situation. To write and produce a book of this dimension must have meant exhaustion and impatience in the end. But this book in its majority, is a great effort bordering on a masterpiece and is a must for plastic surgeons who want to look at themselves in the mirror, as well as for libraries around the world.

